# An Exact Formula for Calculating Inverse Radial Lens Distortions

**DOI:** 10.3390/s16060807

**Published:** 2016-06-01

**Authors:** Pierre Drap, Julien Lefèvre

**Affiliations:** Aix-Marseille Université, CNRS, ENSAM, Université De Toulon, LSIS UMR 7296, Domaine Universitaire de Saint-Jérôme, Bâtiment Polytech, Avenue Escadrille Normandie-Niemen, Marseille 13397, France; Julien.Lefevre@univ-amu.fr

**Keywords:** radial distortion, distortion correction, power series

## Abstract

This article presents a new approach to calculating the inverse of radial distortions. The method presented here provides a model of reverse radial distortion, currently modeled by a polynomial expression, that proposes another polynomial expression where the new coefficients are a function of the original ones. After describing the state of the art, the proposed method is developed. It is based on a formal calculus involving a power series used to deduce a recursive formula for the new coefficients. We present several implementations of this method and describe the experiments conducted to assess the validity of the new approach. Such an approach, non-iterative, using another polynomial expression, able to be deduced from the first one, can actually be interesting in terms of performance, reuse of existing software, or bridging between different existing software tools that do not consider distortion from the same point of view.

## 1. Introduction

**Figure 1:**
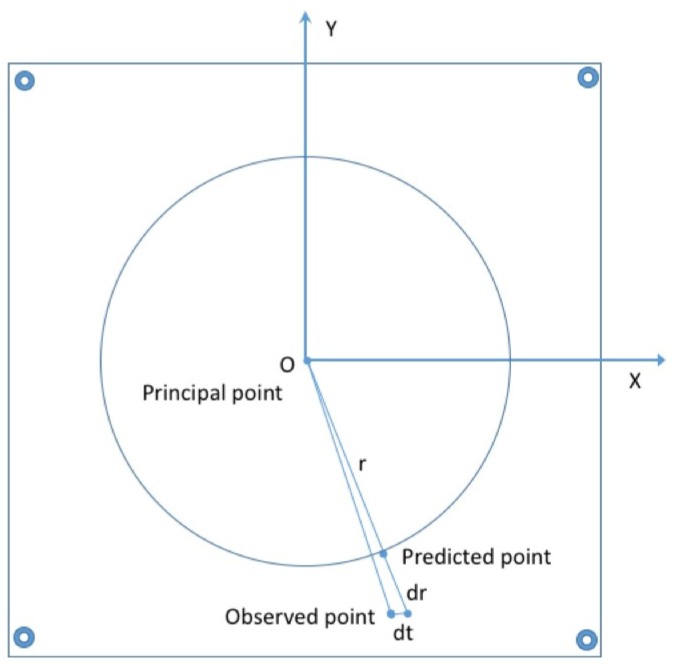
Point shifted by distortion.

Distortion is a physical phenomenon that in certain situations may greatly impact an image’s geometry without impairing quality nor reducing the information present in the image. Applying the projective pinhole camera model is often not possible without taking into account the distortion caused by the camera lens. This phenomenon can be modelled by a radial distortion, the most prominent component, and a second, with a lesser effect, a decentering distortion which has both a radial and a tangential components. Radial distortion is caused by the spherical shape of the lens, whereas tangential distortion is caused by the decentering and non-orthogonality of the lens components with respect to the optical axis ([1,2]). It is important to note that radial distortion is highly correlated with focal length [3] even if in literature it is not modelled within the intrinsic parameters of the camera [4]. This is due to the fact that the radial distortion model is not linear, in contrary to other intrinsic parameters. We can see in Figure 1 the displacement applied to a point caused by both radial and tangential distortion.

Decentering distortion was modelled by Conrady in 1919 [5] then remodelled by Brown in 1971 [6] and a radial distortion model was proposed by Brown in 1966 [7]. These distortion models have been adopted by the Photogrammetry as well as the Computer Vision communities for several decades. Most photogrammetric software such as PhotoModeler (EOS) uses these models (see Equations (1) and (2)) to correct observations visible on the images and provide ideal observations.

**Figure 2:**
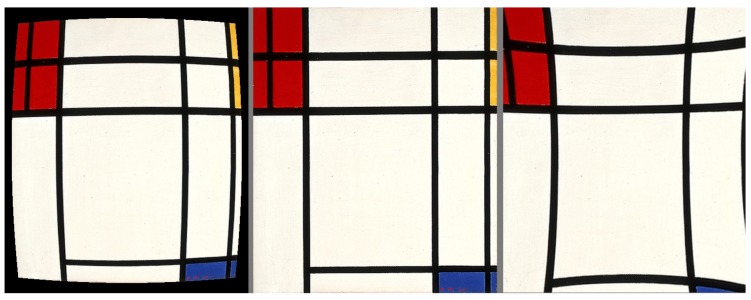
In the center, a painting from Piet Mondrian [12] (which is now in the public domain since 1 January 2016); on the left, the painting with a barrel effect; and on the right, the same image with pincushion distortion.

Roughly, radial distortion can be classified in two families, barrel distortion and pincushion radial distortion. Regarding the $k_{1}$ coefficient in Formula (1), barrel distortion corresponds to a negative value of $k_{1}$ and pincushion distortion to a positive value of $k_{1}$, for an application of the distortion and not a compensation. As shown in Figure 2, barrel and pincushion distortions have an inverse effect and an image affected by a pincushion distortion can be corrected by a barrel distortion (and vice-versa) [8,9,10,11].

Barrel distortion can be physically present in small focal length systems, while larger focal lengths can result in pincushion distortion [8,10]. These radial distortion effects can be very important, especially in inexpensive wide-angle lenses which are often used today.

Using these models to compensate the observations is now well known and many software dealing with images or panoramas propose plugins dedicated to distortion correction (mainly only radial distortion) [13] . However, although we have the equations to compensate the distortion, how to compute the inverse function in order to apply such a distortion is not obvious. For example, when an image of a known 3D point is computed using a calibrated camera, the 2D projected point can be easily computed, but we need then to apply the distortion to the image point in order to obtain an accurate projection of the original 3D point. This first application justifies the present work. How to determine the inverse of a closed form solution for distortion model equations? The second reason is the merging the work of the two communities involved, photogrammetry and computer vision. While having worked separately for years, the situation between the two communities has drastically changed for almost a decade [14]. This is also visible in the form of new commercial or open-source software dealing with photogrammetry or computer vision. For example, PhotoScan (from Agisoft) or MATLAB that comes with the camera calibration toolbox or, for the open-source side, OpenCV toolbox which provides a solution for multiview adjustment. These three software use the same Equations (1) and (2) to manage distortion, but the mathematical model here is used to apply distortion and not to compensate it. Determining an exact formula to calculate inverse lens distortion, which allows using the same software to apply and compensate distortion with two set of $k_{n}$ parameters can be very useful and, in fact, is the purpose of this work.

This paper is organized as follows: in the next section, a demonstration on computing an exact formula for inverse radial distortion is presented. This approach gives a set of $k_{1}...k_{n}$ coefficients computed from the original polynomial distortion model. In Section 3, several applications of this formula are presented. First of all an experiment on this inversion formula is done using only the $k_{1}...k_{n}$ coefficient. Then, an application used to compute a formula for an inverse radial lens distortion is applied to an image coming from a metric camera (Wild P32) with a 3-micron distortion in the edge of the frame. The next experiment is done on a calibration grid built with black disk. Finally, a discussion on converting the distortion model between several photogrammetric software, PhotoModeler (EOS [15]), PhotoScan (Agisoft [16]) and OpenCV [17] is proposed.

## 2. Previous Work

### 2.1. Calibration Approach

Radial distortion is mainly considered in the camera calibration process. Since Duane Brown’s first publication, a large quantity of work was performed in the field of camera calibration, opening the way for new methods. Several techniques were proposed using orthogonal planes, 2D objects with plannars patterns up to self-calibration with unknown 3D points. Interesting reviews were published on both the photogrammetry and computer vision sides by Fraser [18], Zhang [19] and more recently by Shortis [20].

When Brown [6] proposed a radial distortion model in 1971, he also proposed a way to calibrate cameras using a set of plumb lines. The idea of using a set of straight wires to compute a distortion model in a camera calibration process remains in use 45 years later in the fields of photogrammetry and computer vision; Hartley in 1993 [21,22] then Faugeras and Devernay [23] and recently Nomura [24], Clauss [25], Tardif [26], and Rosten [27].

### 2.2. Inverse Radial Distortion

After Conrady and Brown a lot of work was done to deal with removing distortion from images. As the problem is shared by photogrammetry community as well as computer vision we can refer to many books and papers on this topic. Including the famous Manual of photogrammetry [28] and Atkinson gives an overview of these problems for the two communities [29].

Nevertheless, the problem of reverse distortion is somewhat the poor relation of the problems of distortion. As mentioned by Heikkilä and Silvén [30], “only a few solutions to the back-projection problem can be found in literature, although the problem is evident in many applications.” And in the same paper, “we can notice that there is no analytic solution to the inverse mapping”.

In the particular case of high distortion as in wide-angle and fish-eye lenses, some non polynomial (and invertible) models have been proposed; for example Basu and Licardie introduced the Fish-Eye Transform (FET) in [31]. Also Faugeras and Devernay [23] propose another invertible model based on the Field-of-View. A complete description of these models can be found in the review written by Hugues [32] and also in [33].

Regarding the polynomial model, several solutions have been tested to perform inverse radial distortion and the solution can be classified in three main classes (even if other approaches can be found such as the use of a neural network [34]):

- Approximation. Mallon [35], Heikkilä [30,36] and then Wei and Ma [37] proposed inverse approximations of a Taylor expansion including first order derivatives. According to Mallon and Welhan, “This is sometimes assumed to be the actual model and indeed suffices for small distortion levels.” [35]. A global approach, inverse distortion plus image interpolation, is presented in a patent held by Adobe Systems Incorporated [38].
- Iterative. Starting from an initial guess, the distorted position is iteratively refined until a convenient convergence is determined [39,40,41];
- Look-up table. All the pixels are previously computed and a look-up table is generated to store the mapping (as for example in OpenCV).

All these methods involve restrictions and constraints on accuracy, time processing or memory usage.

**Figure 3:**
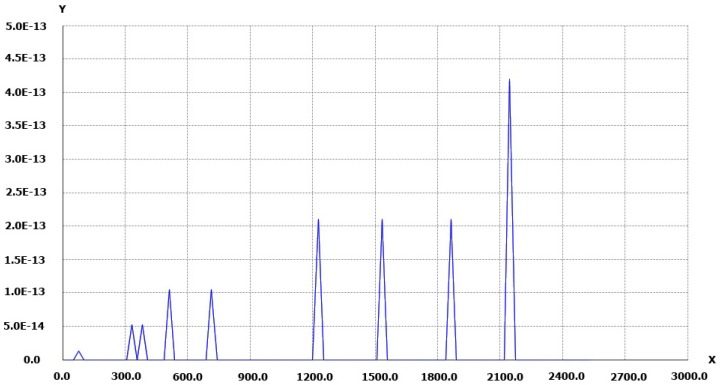
Iterative method applied to a Nikon D700 camera with a 14mm lens. Along the frame diagonal the points are first compensated by what is a normal process of the radial distortion using Equation (1) and then the distortion is applied with the iterative process [40] and the result compared to the original point. On Y-axis, the distance between the original point and the computed reverse point.

Nevertheless, some very good results can be obtained. For example, implementing the iterative approach gives excellent results, however the processing time is drastically increased. Given in Peter Abeles’ blog [40], the method is easy to implement. Results are shown in Figure 3.

The iterative solution works by first estimating the radial distortion magnitude at the distorted point and then refining the estimate until it converges.

Algorithm 1 shows an implementation of this approach.

**Algorithm 1** Iterative algorithm to compute the inverse distortion**Require:** point $P_{n}$ $P_{c}=P_{n}$ **repeat**  $r=\left|\right|P_{c}\left|\right|$  $dr=1+k_{2}r^{2}+k_{4}r^{4}+...$  $P_{c}=P_{n}/dr$ **until** Convergence of $P_{c}$ **return**
$P_{c}$

Only a few iterations are necessary.

The results presented in Figure 3 are in pixels. The used camera here is a Nikon D700 with a 14 mm lens. The calibration was done with PhotoModeler EOS and the results are $k_{1}$ = $1.532\times 10^{-4}$, $k_{2}$ = $-9.656\times 10^{-8}$ , $k_{3}$ = $7.245\times 10^{-11}$. The coefficients are expressed in millimeters. The center of autocollimation and the center of distortion are close to the image center. Only a few iterations are necessary to compute the inverse distortion. In this case, with a calibration made using PhotoModeler [15], the inverse of distortion represents the application of the distortion to a point projected from the 3D space onto the image.

This iterative approach is very interesting when the processing time is not an issue, as for example in the generation of a look-up table. Note, however, that a good initial value is needed.

According to these existing methods, we now want to obtain a formula for the inverse radial distortion when modelled by a polynomial form as described by Brown. The inverse polynomial form will be an expression of the original $k_{1}...k_{4}$ coefficients of the original distortion.

## 3. Exact Formula for Inverse Radial Distortion: An Original Approach

### 3.1. Lens Distortion Models

We consider the general model of distortion correction or distortion removal that can be written in the following form by separating radial and tangential/decentering components:

(1) $$\begin{array}{c} x^{\prime }=x+\bar{x}\left(k_{1}r^{2}+k_{2}r^{4}+k_{3}r^{6}+...\right)+\left[p_{1}\left(r^{2}+2{\bar{x}}^{2}\right)+2p_{2}\bar{x}\bar{y}\right]\left(1+p_{3}r^{2}+...\right) \end{array}$$

(2) $$\begin{array}{c} y^{\prime }=y+\underset{\mathrm{Radial}\mathrm{Distorsion}}{\underset{︸}{\bar{y}\left(k_{1}r^{2}+k_{2}r^{4}+k_{3}r^{6}+...\right)}}+\underset{\mathrm{Tangential}\mathrm{Distorsion}}{\underset{︸}{\left[p_{2}\left(r^{2}+2{\bar{y}}^{2}\right)+2p_{1}\bar{x}\bar{y}\right]\left(1+p_{3}r^{2}+...\right)}} \end{array}$$

where $\bar{x}=x-x_{0}$, $\bar{y}=y-y_{0}$, $r=\sqrt{{\bar{x}}^{2}+{\bar{y}}^{2}}$.

In the following we will consider only radial distortion.

### 3.2. General Framework

Given a model of distortion or correction with parameters $\left(k_{1},k_{2},k_{3},...\right)$, our general objective is to find the inverse transformation. A natural assumption is to express the inverse transformation on the same form of the direct transformation, *i.e.*, with parameters $\left(k_{1}^{\prime },k_{2}^{\prime },k_{3}^{\prime },...\right)$. Therefore we want to express each $k_{i}^{\prime }$ as a function of all the $k_{j}$.

#### Radial distortion

Let us assume that there exists two transformations $T_{1}$ and $T_{2}$:

(3) $$\begin{array}{c} T_{1}:\left(\begin{array}{c} x\\ y \end{array}\right)⟶\left(\begin{array}{c} x^{\prime }\\ y^{\prime } \end{array}\right)=P\left(r\right)\left(\begin{array}{c} x\\ y \end{array}\right) \end{array}$$

(4) $$\begin{array}{c} T_{2}:\left(\begin{array}{c} x^{\prime }\\ y^{\prime } \end{array}\right)⟶\left(\begin{array}{c} x\\ y \end{array}\right)=Q\left(r^{\prime }\right)\left(\begin{array}{c} x^{\prime }\\ y^{\prime } \end{array}\right) \end{array}$$

where $r=\sqrt{x^{2}+y^{2}}$, $r^{\prime }=\sqrt{x^{\prime 2}+y^{\prime 2}}$, and *P* and *Q* are power series:

(5) $$\begin{array}{c} P\left(r\right):=\sum_{n=0}^{+\infty }a_{n}r^{2n} \end{array}$$

(6) $$\begin{array}{c} Q\left(r^{\prime }\right):=\sum_{n=0}^{+\infty }b_{n}r^{\prime 2n} \end{array}$$

with $a_{0}=1$, $a_{1}=k_{1}$,..., $a_{n}=k_{n}$ (in order to use *k* as an index in the calculus of the Appendix). In addition to starting at n=0 we facilitate those calculi. We can scale $r^{\prime }$ as $r^{\prime }=\alpha r$ in order to have the same domain of definition for *P* and *Q*. So *Q* reads:

(7) $$Q\left(r^{\prime }\right)=\sum_{n=0}^{+\infty }b_{n}^{\prime }r^{2n}$$

with $b_{n}^{\prime }=b_{n}\alpha^{2n}$. In the following $b_{n}$ is used instead of $b_{n}^{\prime }$ but we keep in mind this change of variable.

Given the definition of *r* and by using transformation $T_{2}$ in Equation (4) we obtain:

$$r=r^{\prime }\left|Q\left(r^{\prime }\right)\right|$$

and similarly with Equation (3):

$$r^{\prime }=r\left|P\left(r\right)\right|$$

*P* and *Q* are positive which allows removing the absolute value. Hence by injecting the last equation in the first we get:

$$r=rP\left(r\right)Q\left(rP(r)\right)$$

and at the end:

(8) 1=P(r)Q(rP(r)

It is possible to derive a very general relation between coefficients $a_{n}$ and $b_{n}$ but it is not exactly adapted to real situations where *P* is a polynomial of finite order. Therefore we can derive a slightly simpler relationship in the case where only $a_{1},...,a_{4}$ are given. It is summarized in the following result:

**Proposition 1.**  *Given the sequence $a_{1},...,a_{4}$ it is possible to obtain the recursive relation:*

(9) $$b_{0}=1\;and\;for\;n\geq 0\;\;b_{n}=-\sum_{k=1}^{4}a_{k}q\left(n-k\right)-\sum_{\begin{array}{c} j+k=n\\ 0\leq k\\ 1\leq j\leq 8k \end{array}}b_{k}p\left(j,2k\right)$$

*where we use the following intermediate coefficients:*

$$p\left(j,k\right)=\sum_{\begin{array}{c} n_{1}+...+n_{k}=j\\ 0\leq n_{i}\leq 4 \end{array}}a_{n_{1}}...a_{n_{k}}$$

$$q\left(k\right)=-\sum_{j=1}^{4}a_{j}q\left(k-j\right)$$

We will derive this expression in Appendix A and show how the coefficients $b_{1},...,b_{n}$ can be computed both with symbolic and numeric algorithms in Appendix B.

Several remarks can be made about this result:

#### Remark 1

The problem is symmetric in terms of *P* and *Q*, so the relations found for $a_{n}$ can of course be applied in the reverse order.

#### Remark 2

For any *n* the coefficient $b_{n}$ can be computed recursively. In Equation (9), the first summation is obtained thanks to $a_{0},...,a_{4}$ and q(n−1),...,q(n−4) that only depends on the sequence $a_{n}$. Similarly the second summation involves $b_{0},...,b_{n-1}$ and values p(j,2k) which both depend only the given sequence $a_{n}$.

Therefore the recursive formula for $b_{n}$ can be implemented at any order *n*. We provide the 4 first terms:

(10) $$\begin{array}{cc} b_{1} & =-a_{1} \end{array}$$

(11) $$\begin{array}{cc} b_{2} & =3a_{1}^{2}-a_{2} \end{array}$$

(12) $$\begin{array}{cc} b_{3} & =8a_{1}a_{2}-12a_{1}^{3}-a_{3} \end{array}$$

(13) $$\begin{array}{cc} b_{4} & =55a_{1}^{4}+10a_{1}a_{3}-55a_{1}^{2}a_{2}+5a_{2}^{2}-a_{4} \end{array}$$

All formula till $b_{9}$ are summarized in Appendix C.

## 4. Results and Experimental Section

In this section we propose three experiments to test this inverse formula for radial distortion in order to evaluate the relevance of such approach.

- First, we begin by testing the accuracy of the inverse formula by applying the forward/inverse formula recursively within a loop. Hence, the inverse of the inverse radial distortion is computed 10,000 times and compared to the original distortion coefficients.
- Then, for a given calibrated camera, we compute the residual after applying and compensating the distortion along the camera frame. A residual curve shows the results of the inverse camera in all the frame.
- The last experiment is the use of inverse distortion model on a image made with a metric camera built with a large eccentricity (a film-based Wild P32 camera), without distortion. We apply a strong distortion and then compensate it and finally compare it to the original image.

### 4.1. Inverse Distortion Loop

In this experiment, as the formula gives an inverse formula for radial distortion we do it twice and compare the final result with the original one. In a second step, we iterate this process 10,000 times and compare the final result with the original distortion.

The Table 1 shows the original radial distortion and the computed inverse parameters. The original distortion is obtained by using PhotoModeler to calibrate a Nikon D700 camera with a 14 mm lens from Sigma.

The results for this step are presented in Table 2. In the columns ‘Delta Loop 1’ and ‘Delta Loop 10000’ we can see that $k_{1}$ and $k_{2}$ did not change and the delta on $k_{3}$ and $k_{4}$ are small with respect to the corresponding coefficients: the error is close to 1E-10 smaller than the corresponding coefficient. Note that $k_{4}$ was not present in the original distortion and as the inverse formula is in function of only $k_{1}...k_{4}$, the loop is computed without the coefficients $k_{5}...k_{9}$ which influences the results, visible from $k_{4}$.

This first experiments shows the inverse property of the formula and of course not the relevance of an inverse distortion model. But this experiment shows also the high stability of the inversion process. However, even if coefficients $k_{1}...k_{4}$ are sufficient in order to compensate distortion, the use of coefficients $k_{1}...k_{9}$ are important for the inversion stability.

The next two experiments show the relevance of this formula for the inverse radial distortion model.

### 4.2. Inverse Distortion Computation onto a Frame

This second experiment uses a Nikon D700 equipped with a 14 mm lens from Sigma. This camera is a full frame format, *i.e.*, a 24 mm × 36 mm frame size. The camera was calibrated using PhotoModeler and the inverse distortion coefficients are presented in Table 1, where Column 1 gives the calibration result on the radial distortion, and Column 2 the computed inverse radial distortion.

Note that the distortion model provided by the calibration using PhotoModeler gives as a result a compensation of the radial distortion, in millimeters, limited to the frame.

The way to use this coefficient is to first express a 2D point on the image in the camera reference system, in millimeters, with the origin on the CoD (Center of Distortion), close to the center of the image. Then the polynomial model is applied from this point.

The inverse of this distortion is the application of such a radial distortion to a point theoretically projected onto the frame.

In all following experiments, the residuals are computed as follows:

A 2D point $p_{}$, is chosen inside the frame, its coordinate are previously computed in millimeters in the camera reference system with the origin on the CoD. Then $p_{1}$ is $p_{}$ compensated by the inverse of distortion. Finally $p_{2}$ is $p_{1}$ compensated by the original distortion.

The residual is the value $dist(p_{},p_{2})$ .

**Figure 4:**
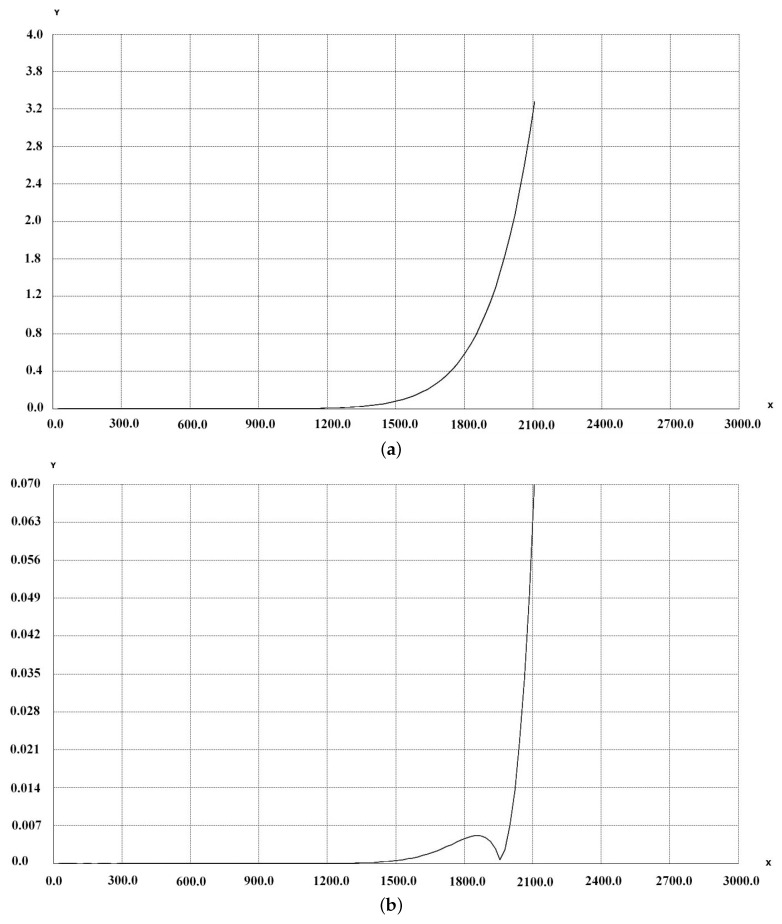
Inverse residual distortion with $k_{1}..k_{9}$ Coefficient Computed for the entire frame. (**a**) Inverse residual distortion with $k_{1}...k_{4}$ Coefficient Computed from 0 to maxX frame; (**b**) Inverse residual distortion with $k_{1}..k_{9}$ Coefficient Computed from 0 to maxX frame.

The following results show the 2D distortion residual curve. For a set of points on the segment [O,maxX/2] the residuals are computed and presented in Figure 4 as Y-axis. The X-axis represents the distance from the CoD. These data comes from the calibration process and are presented in Table 1.

**Figure 5:**
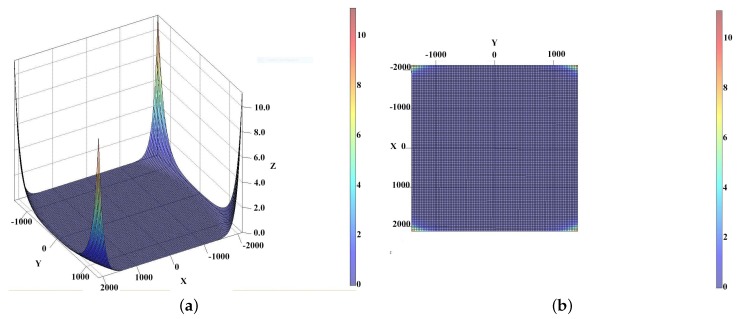
Inverse residual distortion with $k_{1}...k_{9}$ coef. Computed on the entire frame. (**a**) Axonometric view; (**b**) Top view.

The results shown in Figure 4 and Figure 5 are given in pixels.

In Figure 4a, below, we present the residuals using only coefficients $k_{1}..k_{4}$ of the inverse distortion. The maximum residual is close to 4 pixels, but residuals are less than one pixel until close to the frame border. This can be used when using non configurable software where it is not possible to use more than 4 coefficients for radial distortion modeling.

In Figure 4, below, we present the residual computed from 0 to maxX frame using coeficients $k_{1}...k_{9}$ for inverse distortion.

The results are very good, less than 0.07 pixel on the frame border along 0X axis and the performance is quite the same as for compensating the original distortion.

We can see that in almost all the images the residuals are close to the ones presented in Figure 5. Nevertheless, we can observe in Figure 4 higher residual in the corners, where the distance to the CoD is the greatest.

Here follows a brief analysis of the residuals:

These two experiments show that the results are totally acceptable even if the residuals are higher in zone furthest from the CoD, *i.e.*, in the diagonal of the frame. As shown in Table 3, only 2.7 % of the frames have residuals > 1 pixel.

### 4.3. Inverse Distortion Computation on an Image Done with a Metric Camera

This short experiment used an image taken with a Wild P32 metric camera in order to work on an image without distortion. The Wild P32 terrestrial camera is a photogrammetric camera designed for close-range photogrammetry, topography, architectural and other special photography and survey applications.

This camera was used as film based, the film is pressed onto a glass plate fixed to the camera body on which 5 fiducial marks are incised. The glass plate prevents any film deformation.

**Figure 6:**
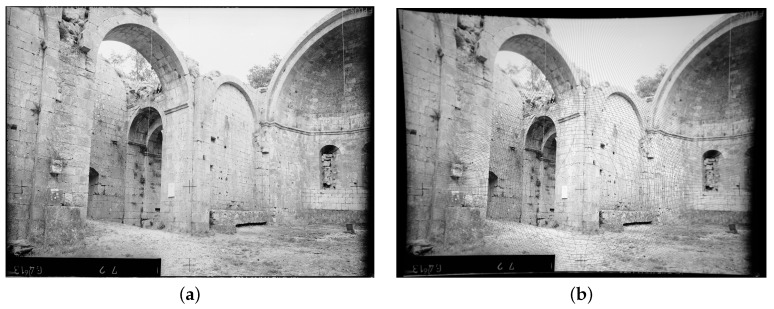
Distortion-less image taken using a small-format Wild P32 metric camera and application of an artificial distortion. (**a**) On the left, original image taken with P32 Wild metric camera; (**b**) On the right, pincushion distortion applied on this original image whithout interpolation. As images have not the same pixel size some vacant pixel are visible as black lines (see Hughues [32]). These lines surround the distortion center, here located on a fiducial mark, strongly shifted from the image center.

The film format is 65 mm × 80 mm and the focal length, fixed, is 64 mm. Designed for architectural survey the camera has a high eccentricity and the 5 fiducial marks were used in this paper to compute the CoD. In Figure 6a four fiducial marks are visible (the fifth is overexposed in the sky). The fiducial marks are organized as follows: one at the principal point (PP), three at 37.5 mm from the PP (left,right,top) and one at 17.5 mm (bottom).

This image was taken in 2000 in the remains of the Romanesque Aleyrac Priory, in northern Provence (France) [42]. Its semi-ruinous state gives a clear insight into the constructional details of its fine ashlar masonry as witnessed by this image taken using a Wild P32 during a photogrammetric survey.

As this image did not have any distortion, we used a polynomial distortion coming from another calibration and adapted it to the P32 file format (see Table 4). The initial values of the coefficients have been conserved and the distortion polynom expressed in millimeters is the compensation due at any point of the file format. The important eccentricity of the CoD is used in the image rectification: the COD is positioned on the central fiducial marks visible on the images in Figure 6a,b.

After scanning the image (the film was scanned by Kodak and the result file is a 4860 × 3575 pixel image), we first measure the five fiducial marks in pixels on the scanned image and then compute an affine transformation to pass from the scanned image in pixels to the camera reference system in millimeters where the central cross is located at (0.0, 0.0). This is done according to a camera calibration provided by the vendor, which gives the coordinates of each fiducial mark in millimeters in the camera reference system. Table 5 shows the coordinates of the fiducial marks and highlights the high eccentricity of the camera built for architectural survey. This operation is called internal orientation in photogrammetry and it is essential when using images coming from film-based camera that were scanned. The results of these measurements are shown in Table 5.

**Figure 7:**
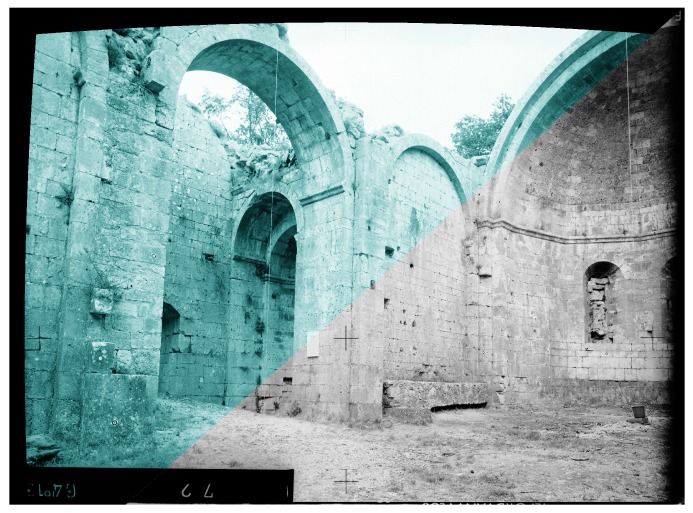
Distortion compensation applied on the pincushion image obtained in Figure 6b. In green the image corrected by inverse distortion; in black and white the original image.

In Figure 6a we can see the original image taken in Aleyrac while in the Figure 6b we can see the result of the radial distortion inversion. Figure 7 shows the original image in grey and the image computed after a double inversion of the radial distortion model in green.

We can observe no visible difference in the image. This is correlated with the previous results in the second experiment, see Figure 4.

## 5. Conclusions and Discussion

The experiments presented in this article show the relevance of the proposed methodology and the reliability of the result. However, a significant difference exists depending on whether the set of coefficients $k_{1}...k_{4}$ or $k_{1}...k_{9}$. For large distortion the number of parameters should be significant. See Figure 4 and Figure 5 for the influence of the number of coefficients. We can note that since the formulation by Brown, the number of coefficients used to characterize the distortion has increased. In 2015 the Agisoft company added $k_{4}$ in their radial distortion model while at the same time many software still use only $k_{1}$, $k_{2}$ and in 2016 they add $p_{3}$ and $p_{4}$ to the tangential distortion model.

Even when $k_{1}...k_{4}$ are sufficient for compensating the radial distortion, it is however necessary to increase the degree of the polynomial to correctly compute the inverse.

### 5.1. A Bridge between PhotoModeler and Agisoft for Radial Distortion

One of the applications for using such a formula to compute the inverse distortion coefficient in function of $k_{1}...k_{4}$ is to convert distortion models between two software programs that use the inverse distortion model, as for example PhotoModeler and PhotoScan from Agisoft. Indeed PhotoModeler uses the Brown distortion model to compensate for observations made on images and so to obtain a theoretical observation without distortion effect. In contrary, PhotoScan from Agisoft uses a similar model but it adds the distortion to a point projected onto the image. To convert a distortion model from PhotoModeler to PhotoScan, or vice versa, we need to compute the inverse distortion model. We need to take in consideration the unit used to express the 2D point coordinate: in PhotoModeler the points are measured in millimeters and their range is limited to the camera frame; whereas in PhotoScan, the points are normalized by the focal length.

To convert a distortion model from PhotoModeler to PhotoScan the following steps are necessary:

1. Given $k_{1}...k_{3}$ as the coefficients of the polynom modeling the radial distortion in PhotoModeler. Note that PhotoModeler uses only $k_{1}...k_{3}$ coefficient.
2. $k_{1}=k_{1}*focalmm^{2}$
  $k_{2}=k_{2}*focalmm^{4}$
  $k_{3}=k_{3}*focalmm^{6}$
3. Compute $k_{1}^{\prime }...k_{4}^{\prime }$ (PhotoScan uses $k_{4}$) according to Appendix C.

And to obtain the $k_{1}...k_{3}$ for PhotoModeler starting from $k_{1}..k_{4}$ given by PhotoScan we need:

1. Given $k_{1}...k_{4}$ as the coefficient of the polynom modeling the radial distortion in PhotoScan.
2. $k_{1}=k_{1}/focalmm^{2}$
  $k_{2}=k_{2}/focalmm^{4}$
  $k_{3}=k_{3}/focalmm^{6}$
  $k_{4}=k_{4}/focalmm^{8}$
3. compute $k_{1}^{\prime }...k_{3}^{\prime }$ (PhotoModeler uses $k_{3}$) according to Appendix C.

The proposed approach in this article allows to compute the new coefficients in function of $k_{1}...k_{4}$

### 5.2. Possible Limitations

Our results on inverse residual distortion suggests a decrease with the order of approximation. In the future it could be interesting to determine some analytical bounds on the maximal residual distortion. This bound would depend on the distortion coefficients $k_{1},k_{2},k_{3},k_{4}$, maxX and the order *N*. The crucial question would be to know whether this bound converges when *N* goes to infinity. This is absolutely not guaranteed since the formula of proposition 1 has been obtained by purely formal manipulations and the power series *Q* could be divergent (which implies the divergence of the residual). In this case, one could however expect good behavior, similarly to formal solutions of differential equations, whose approximations can be controled for r<R until a bound *N* depending on *R* [43] (It is also explained more briefly in [44], Section 3 page 103). At a given *N* one could also expect that this bound will decrease if the distortion given by *P* is decreased.

We thought such technical questions may be of great interest, both from a mathematical perspective as well as an applied one. Obtaining theoretical results on the inverse residual distortion might influence the software community in adding more coefficients in the polynomial models.

## Figures and Tables

**Table 1:** Radial distortion calibration in Column two. Column three, inverse of radial distortion with coefficient from $k_{1}...k_{9}.$

Radial Distortion Coefficients	Original Value	Computed Inverse Value
$k_{1}$	$1.532\times 10^{-4}$	$1.532\times 10^{-4}$
$k_{2}$	$-9.656\times 10^{-8}$	$1.6697072\times 10^{-7}$
$k_{3}$	$7.245\times 10^{-11}$	$-2.33941625216\times 10^{-10}$
$k_{4}$	0.0	$3.1255518770316804\times 10^{-13}$
$k_{5}$	0.0	$-4.774156462972984\times 10^{-16}$
$k_{6}$	0.0	$7.680785197322419\times 10^{-19}$
$k_{7}$	0.0	$-1.1582853960835112\times 10^{-21}$
$k_{8}$	0.0	$2.1694555835054252\times 10^{-24}$
$k_{9}$	0.0	$-3.779164309884112\times 10^{-27}$

**Table 2:** Radial distortion inverse loop and residual between coefficients of the orignal distortion and after *n* inversions (*n* = 2 and *n* = 10,000).

Coefficient	Original	Inverse 1	Delta Loop 1	Delta Loop 10,000
$k_{1}$	$1.532\times 10^{-4}$	$-1.532\times 10^{-4}$	0.0	0.0
$k_{2}$	$-9.656\times 10^{-8}$	$1.6697072\times 10^{-7}$	0.0	0.0
$k_{3}$	$7.245\times 10^{-11}$	$-2.33941625216\times 10^{-10}$	$1.292469707114\times 10^{-26}$	$1.292469707\times 10^{-26}$
$k_{4}$	0.0	$3.1255518770316804\times 10^{-13}$	$1.009741958682\times 10^{-28}$	$1.009842932\times 10^{-24}$

**Table 3:** Residuals on the full frame format.

Pixel by Residual	Nb Concerned Pixel
Pixels	10,000.0
Pixels with residual < 0.2	9344.0 (93.44 %)
Pixels with residual < 1.0	9732.0 (97.32 %)
Pixels with residual > 1.0	268.0 (2.68 %)

**Table 4:** Radial distortion comensation and then application of the inverse used with the image taken with the P32 camera.

Coef.	Original	Inverse
$k_{1}$	0.09532	−0.09532
$k_{2}$	$-9.656\times 10^{-8}$	0.02725780376
$k_{3}$	$7.245\times 10^{-11}$	−0.010392892306459602
$k_{4}$	0.0	0.004540497555744342
$k_{5}$	0.0	−0.0021482705738196948
$k_{6}$	0.0	0.0010711249019932042
$k_{7}$	0.0	$-5.542464764540273\times 10^{-4}$
$k_{8}$	0.0	$2.948490225469636\times 10^{-4}$
$k_{9}$	0.0	$-1.6024842649677896\times 10^{-4}$

**Table 5:** Photograph taken with the Wild P32 camera: data and some results of the Internal Orientation.

Param.	X Value	Y Value
mm 2 Pixel	58.885	58.885
Frame size in pixel	4860	3575
Frame size in mm	82.54	60.71
CoD, ppx ppy (pixel)	2423.212	2377.528
Fiducial mark-up- (mm)	0.0	37.5
Fiducial mark-right- (mm)	37.5	0.0
Fiducial mark-down- (mm)	0.0	−17.5
Fiducial mark-left- (mm)	−37.5	37.5
Fiducial mark-center- (mm)	0.0	0.0

## Appendix A

In Equation (8) we replace *P* and *Q* by their power series in order to identify coefficients

$$1=\sum_{m=0}^{+\infty }a_{m}r^{2m}\sum_{n=0}^{+\infty }b_{n}{\left(rP(r)\right)}^{2n}$$

For *k* fixed $P{\left(r\right)}^{k}$ can be rewritten as a product:

$$P{\left(r\right)}^{k}=\sum_{n_{1}=0}^{4}a_{n_{1}}r^{2n_{1}}...\sum_{n_{k}=0}^{4}a_{n_{k}}r^{2n_{k}}$$

which gives a more compact expression

$$P{\left(r\right)}^{k}=\sum_{m=0}^{4k}r^{2m}\sum_{\begin{array}{c} n_{1}+...+n_{k}=m\\ 0\leq n_{i}\leq 4 \end{array}}a_{n_{1}}...a_{n_{k}}=\sum_{m=0}^{4k}r^{2m}p\left(m,k\right)$$

Note that p(m,k)=0 as soon as m>4k. Then we obtain:

$$Q\left(rP(r)\right)=\sum_{n=0}^{+\infty }b_{n}r^{2n}\sum_{m=0}^{8n}r^{2m}p\left(m,2k\right)$$

$$Q\left(rP(r)\right)=\sum_{k=0}^{+\infty }r^{2k}\underset{t(k)}{\underset{︸}{\sum_{\begin{array}{c} m+n=k\\ 0\leq n\\ 0\leq m\leq 8n \end{array}}b_{n}p\left(m,2n\right)}}$$

Let us call t(k) the coefficient in the previous sum. Actually t(k) will turn out to be q(k), defined in Proposition 1. Last we can express the initial equality $P\left(r\right)Q\left(r(P(r)\right)=1$:

$$\sum_{k=0}^{+\infty }a_{k}r^{2k}\sum_{j=0}^{+\infty }r^{2j}t\left(j\right)=1$$

$$\sum_{l=0}^{+\infty }r^{2j}\sum_{k+j=l}a_{k}t\left(j\right)=1$$

To obtain the equality:

$$\sum_{k=0}^{l}a_{k}t\left(l-k\right)=0\mathrm{for}l\geq 1$$

We decompose this sum:

$$a_{0}t\left(l\right)+\sum_{k=1}^{l}a_{k}t\left(l-k\right)=0$$

and since $a_{0}=1$ we conclude that t(l) satisfy the same recurrence relationship as *q* in Proposition 1. The two quantities are therefore equal since they have the same initial value. We have also an alternative expression for *q* given by the definition of *t* depending on $b_{n}$ and p(m,2n) that gives:

$$b_{k}p\left(0,2k\right)+\sum_{\begin{array}{c} m+n=k\\ 0\leq n\\ 1\leq m\leq 8n \end{array}}b_{n}p\left(m,2n\right)=t\left(k\right)$$

By remarking that p(0,2k)=1 we obtain Proposition 1.

## Appendix B

There are two ways of implementing the result of Proposition 1. One can be interested in having a symbolic representation of coefficients $b_{n}$ with respect to $a_{1},a_{2},a_{3},a_{4}$. But one can also simply obtain numeric values for $b_{n}$ given numeric values for $a_{n}$.

The main ingredient in both cases is to compute efficiently the coefficients p(j,k). One can start by the trivial result that if $n_{1}+...+n_{k}=j$ then $n_{1}+...+n_{k-1}=j-n_{k}$. But $n_{k}$ can take only 5 values between 0 and 4 (there are no other coefficient $a_{n}$ in our case). Therefore one can easily derive the recursive identity:

$$p\left(j,k\right)=p\left(j,k-1\right)+a_{1}p\left(j-1,k-1\right)+a_{2}p\left(j-2,k-1\right)+a_{3}p\left(j-3,k-1\right)+a_{4}p\left(j-4,k-1\right)$$

To compute $b_{n}$ we need coefficients p(j,2k) with the constraints j+k=n, 0≤k and 1≤j≤8k. A table (n−1)×2(n−1) is defined and coefficients are progressively filled by varying the coefficient *k* from 1 to 2(n−1) thanks to dynamic programming. This step is summarized in Algorithm B1.

**Algorithm B1** Computation of p(j,k)
**Require:** coefficients $a_{1},a_{2},a_{3},a_{4}$, integer *N*
Define an array *p* of size N×2N−1
**for** k=0:2(N−1) **do**
p(0,k)=1
**end for**
**for** k=1:2(N−1) **do**
**for** j=1:2(N−1) **do**
$p\left(j,k\right)=p\left(j,k-1\right)+a_{1}p\left(j-1,k-1\right)+a_{2}p\left(j-2,k-1\right)+a_{3}p\left(j-3,k-1\right)+a_{4}p\left(j-4,k-1\right)$
▹ With p(j,k)=0 as soon as j<0 or k≤0
**end for**
**end for**
**return** *p*

The most tricky aspect is to manipulate formal terms with $a_{1},a_{2},a_{3},a_{4}$. For that it is good to remark that coefficients $b_{n}$ are made of terms $a_{1}^{n_{1}}a_{2}^{n_{2}}a_{3}^{n_{3}}a_{4}^{n_{4}}$ such as $n_{1}+2n_{2}+3n_{3}+4n_{4}=n$. We can therefore have an a priori bound on each exponents, *n*, n/2, n/3, n/4 respectively. Given that, each multinomial term can be represented as a coefficient in a 4D array of bounded size. It is also very convenient to use a sparse representation for it due to many vanishing terms. Addition of terms are simply additions of 4D arrays of size bounded by $n^{4}$. Multiplication requires shifting operations on dimensions of the array, basically multiplying by $a_{1}^{n_{1}}$ corresponds to a translation by $n_{1}$ of the first dimension.

**Algorithm B2** Computation of coefficients $b_{n}$
**Require:** coefficients $a_{1},a_{2},a_{3},a_{4}$, integer *N*
Define an array q=[0,0,0,0]
Define an array *b* of size *N*
b(0)=1
Compute *p* with Algorithm B1
**for** n=1:N **do**
tmp=0
**for** k=1:4 **do**
$tmp=tmp-a_{k}q\left(k-1\right)$
**end for**
b(n)=b(n)+tmp
**for** k=n/9:(n−1) **do**
b(n)=b(n)−b(k)p(n−k,2k)
**end for**
q(1:3)=q(0:2) and q(0)=tmp
**end for**
**return** *b*

We summarize the computation (Algorithm B2).

## Appendix C

Here are the formula for the nine first coefficients $b_{n}$. Note that there are no more coefficients $-k_{n}$ for $b_{n}$, when n≥5, since $k_{n}=0$.

$\begin{array}{cc} b_{1} & =-k_{1} \end{array}$
$\begin{array}{cc} b_{2} & =3k_{1}^{2}-k_{2} \end{array}$
$\begin{array}{cc} b_{3} & =-12k_{1}^{3}+8k_{1}k_{2}-k_{3} \end{array}$
$\begin{array}{cc} b_{4} & =55k_{1}^{4}-55k_{1}^{2}k_{2}+5k_{2}^{2}+10k_{1}k_{3}-k_{4} \end{array}$
$\begin{array}{cc} b_{5} & =-273k_{1}^{5}+364k_{1}^{3}k_{2}-78k_{1}k_{2}^{2}-78k_{1}^{2}k_{3}+12k_{2}k_{3}+12k_{1}k_{4} \end{array}$
$\begin{array}{cc} b_{6} & =1428k_{1}^{6}-2380k_{1}^{4}k_{2}+840k_{1}^{2}k_{2}^{2}-35k_{2}^{3}+560k_{1}^{3}k_{3}-210k_{1}k_{2}k_{3}+7k_{3}^{2}-105k_{1}^{2}k_{4}+14k_{2}k_{4} \end{array}$
$\begin{array}{cc} b_{7} & =-7752k_{1}^{7}+15504k_{1}^{5}k_{2}-7752k_{1}^{3}k_{2}^{2}+816k_{1}k_{2}^{3}-3876k_{1}^{4}k_{3}+2448k_{1}^{2}k_{2}k_{3}-136k_{2}^{2}k_{3}-136k_{1}k_{3}^{2}\\ & +816k_{1}^{3}k_{4}-272k_{1}k_{2}k_{4}+16k_{3}k_{4} \end{array}$
$\begin{array}{cc} b_{8}= & 43263k_{1}^{8}-100947k_{1}^{6}k_{2}+65835k_{1}^{4}k_{2}^{2}-11970k_{1}^{2}k_{2}^{3}+285k_{2}^{4}+26334k_{1}^{5}k_{3}-23940k_{1}^{3}k_{2}k_{3}\\ & +3420k_{1}k_{2}^{2}k_{3}+1710k_{1}^{2}k_{3}^{2}-171k_{2}k_{3}^{2}-5985k_{1}^{4}k_{4}+3420k_{1}^{2}k_{2}k_{4}-171k_{2}^{2}k_{4}-342k_{1}k_{3}k_{4}+9k_{4}^{2} \end{array}$
$\begin{array}{cc} b_{9} & =-246675k_{1}^{9}+657800k_{1}^{7}k_{2}-531300k_{1}^{5}k_{2}^{2}+141680k_{1}^{3}k_{2}^{3}-8855k_{1}k_{2}^{4}-177100k_{1}^{6}k_{3}\\ & +212520k_{1}^{4}k_{2}k_{3}-53130k_{1}^{2}k_{2}^{2}k_{3}+1540k_{2}^{3}k_{3}-17710k_{1}^{3}k_{3}^{2}+4620k_{1}k_{2}k_{3}^{2}-70k_{3}^{3}+42504k_{1}^{5}k_{4}\\ & -35420k_{1}^{3}k_{2}k_{4}+4620k_{1}k_{2}^{2}k_{4}+4620k_{1}^{2}k_{3}k_{4}-420k_{2}k_{3}k_{4}-210k_{1}k_{4}^{2} \end{array}$
